# Women’s economic empowerment, participation in decision-making and exposure to violence as risk indicators for early childhood caries

**DOI:** 10.1186/s12903-020-1045-5

**Published:** 2020-02-17

**Authors:** Morenike Oluwatoyin Folayan, Maha El Tantawi, Ana Vukovic, Robert Schroth, Balgis Gaffar, Ola B. Al-Batayneh, Rosa Amalia, Arheiam Arheiam, Mary Obiyan, Hamideh Daryanavard, Early Childhood Caries Advocacy Group

**Affiliations:** 1 Department of Child Dental Health, Obafemi Awolowo University, Ile-Ife, Nigeria; 2 Department of Pediatric Dentistry and Dental Public Health, Faculty of Dentistry, Alexandria University, Alexandria, Egypt; 3 Department of Pediatric and Preventive Dentistry, School of Dental Medicine, University of Belgrade, Belgrade, Serbia; 4 Department of Preventive Dental Science, Dr. Gerald Niznick College of Dentistry, University of Manitoba, Winnipeg, Canada; 5 Departments of Pediatrics and Child Health and Community Health Sciences, Max Rady College of Medicine, Rady Faculty of Health Sciences, University of Manitoba, Winnipeg, Canada; 6 Department of Preventive Dental Sciences, College of Dentistry, Imam Abdulrahman bin Faisal University, Dammam, Saudi Arabia; 7 Department of Preventive Dentistry, Jordan University of Science and Technology, PO Box 3030, Irbid, 22110 Jordan; 8 Department of Preventive and Community Dentistry, Faculty of Dentistry, Universitas Gadjah Mada Yogyakarta, Yogyakarta, Indonesia; 9 Department of Community and Preventive Dentistry, University of Benghazi, Benghazi, Libya; 10 Department of Demography and Social Statistics, Obafemi Awolowo University, Ile-Ife, Nigeria; 11 Dubai Health Authority, Dubai, United Arab Emirates

**Keywords:** Early childhood caries, Women empowerment, Decision-making, Violence

## Abstract

**Objectives:**

In view of the association between early childhood caries (ECC])and maternal social risk factors, this study tried to determine if there were associations between indicators of processes, outputs and outcomes of women’s empowerment, and the prevalence of ECC.

**Methods:**

In this ecological study, indicators measuring the explanatory variables - economic empowerment, decision-making and violence against women - were selected from the Integrated Results and Resources Framework of the UN-Women Strategic Plan 2018–2021 and WHO database. Indicators measuring the outcome variables - the prevalence of ECC for children aged 0 to 2 years, and 3 to 5 years - were extracted from a published literature. The general linear models used to determine the association between the outcome and explanatory variables were adjusted for economic level of countries. Regression estimates (B), 95% confidence intervals and partial eta squared (η^2^) were calculated.

**Results:**

Countries with more females living under 50% of median income had higher prevalence of ECC for 3 to 5-year olds (B = 1.82, 95% CI = 0.12, 3.52). Countries with higher percentage of women participating in their own health care decisions had higher prevalence of ECC for 0 to 2-year-olds (B = 0.85, 95% CI = 0.03, 1.67). Countries with higher percentage of women participating in decisions related to visiting family, relatives and friends had higher prevalence of ECC for 3 to 5-year-olds (B = 0.67, 95% CI = 0.03, 1.32). None of the indicators for violence against women was significantly associated with the prevalence of ECC.

**Conclusion:**

Empowerment of women is a welcome social development that may have some negative impact on children’s oral health. Changes in policies and norms are needed to protect children’s oral health while empowering women.

## Introduction

Early childhood caries (ECC) is a non-communicable disease with both biological and social constructs. It affects more than 621 million children worldwide [1–3], and negatively impacts the general health and wellbeing of children [4, 5]. There is extensive literature highlighting the biological etiology and risk indicators for ECC [5–7] and a few on the family related risk factors. Maternal factors such as age and marital status [8] psychosocial status [9, 10], knowledge of oral health [10, 11] and oral health behavior [12, 13] are risk factors for ECC. The link between maternal health status and child oral health is well-documented [9, 12, 14].

While there is some clarity on the association between maternal wellbeing and ECC, little is known about how social constructs of maternal wellbeing also affects the risk for ECC. One possible social construct that may affect both maternal wellbeing and the risk of a preschooler to ECC is women’s empowerment status, defined as a mother having power and control over resources and decisions [15]. Several publications reported that maternal empowerment is associated with maternal survival [16–20] and child health outcomes [21, 22]. Empowered mothers are less likely to have malnourished children [23] because they have improved diet quality diversity [24, 25], and support better food consumption [25]. Maternal empowerment is also associated with increased mobility that improves social networking [26, 27], access to health information [16, 28, 29] and access to food and medicines [30, 31].

Women’s empowerment is moderated by maternal education and wealth [16]. Higher maternal education levels and economic status are associated with use of modern health facilities, contraception, preventive care, and reduced smoking [18, 32–34]. It also improves child health outcomes [24, 35–44]. Higher maternal economic status is associated with better residential locations that increase access to health services and clean environment [37, 45, 46]; predictors of infant and child morbidity and mortality [45, 47, 48].

Gender inequalities limit access of women to education, job opportunities with significant impact on their economic status [49]. This is also associated with reduced control of resources by women and the risk for abuse [50]. The concept of empowerment was developed as a holistic process to address this inequity. It acts through modifying individual women’s abilities and personal choices and collective growth achieved by cultural norms [51, 52].

We conceptualize that reduced women economic empowerment decreases their decision-making ability, thereby decreasing female agency (i.e, the ability to make things happen) [53, 54]. With decreased agency, the risk of exposure to and tolerance of violence and abuse would increase [55–57]. This impacts women in ways that reduces the ability of mothers to make health choices for the child [58–61].

In the absence of accessible evidence on the relationship between women empowerment and ECC we designed this study to determine if there is an association between women’s empowerment, decision-making status, exposure to violence, and the prevalence of ECC. We hypothesized that the higher proportion of empowered women, the lower the prevalence of ECC.

## Methods

**Fig. 1:**
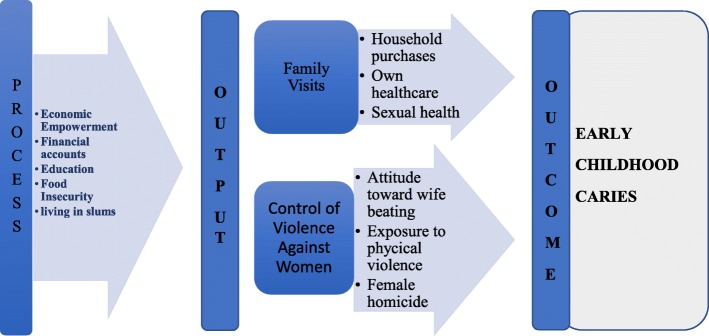
The contextual framework of women empowerment process and output factors that play a role in early childhood caries outcome

Country-level data on the prevalence of ECC and women economic empowerment, decision-making and exposure to violence reported in the (Appendix A), were used for this ecological study. Figure 1 is a representation of the framework guiding the selection of indicators for this study wherein economic empowerment, decision-making ability and less exposure to violence reflect improved women’s well-being that is associated with lower risk for ECC [62, 63]. The framework was developed based on the Integrated Results and Resources Framework of the United Nations-Women Strategic Plan 2018–2021 [64]. This document included a set of indicators used to measure country progress toward women empowerment. We selected indicators measuring national individual-based performance relative to the total population (percentages) as these were more appropriate to study the micro-level relationship between women’s status and ECC. Indicators related to governments or national guidelines were not used since they imply a macro-level perspective that is beyond the scope of the current study. Mean values for the years 2009 to 2017 were computed.

### Data on economic empowerment status

Women’s economic empowerment status was measured using five variables: owning a financial account, completing secondary school education, food insecurity, living in slums, and having an income equal to or above 50% of the median income for the country. Table 1 shows the definitions of these variables.

**Table 1:** Definition and data sources of the indicators of women empowerment

Variables	Definition
ECONOMIC EMPOWERMENT	
Education	The percentage of female population 25 years and older who attained or completed secondary education. This data was obtained from the World Bank Databank Gender Statistics [65], based on the Global Findex database
Food insecurity	The percentage of females who have experienced food insecurity at moderate and severe levels during the 12 months reference period. This information was obtained from the 2014–15 Food Insecurity Experience Scale survey [66].
Living in slums	The proportion of women 15–49 years old who live in households that meet at least one of 5 criteria: (1) lack of access to improved water source, (2) lack of access to improved sanitation facilities, (3) lack of sufficient living area, (4) lack of housing durability and (5) lack of security of tenure. This was obtained from the DHS of 60 developing countries as reported in the UN Women Report [67].
Below 50% of median income	The percentage of females living below 50% of the median income in the country. Income is defined as total monetary payments from labor, property, and social or private transfers. It also includes total value of non-monetary goods and services received from labor and social or private transfers, excluding social transfers in kind such as universal health insurance, universal education benefits, and near cash benefits from public housing. Data was derived from the most recent Luxembourg Income Study datasets [68].
Financial account	The percentage of female respondents 15 years and above, who report having their own account or together with someone else at a bank or another type of financial institution. This data was obtained from the World Bank Databank Gender Statistics [69], based on the Global Findex database
WOMEN’S DECISION-MAKING ABILITY	
Family visits	The percentage of currently married women aged 15–49 years who say that they alone or jointly have the final say in visits to family, relatives, and friends [65].
Household purchase decisions	The percentage of currently married women aged 15–49 years who say that they alone or jointly have the final say in making major household purchases [65].
Own health care	The percentage of currently married women aged 15–49 years who say that they alone or jointly have the final say in own health care [65].
Sexual health	The proportion of women ages 15–49 years (married or in union) who can say no to sexual intercourse with their husband or partner if they do not want, decide on use of contraception, and decide on their reproductive health care. Only women who provide a “yes” answer to all three components are considered as women who “make her own decisions regarding sexual and reproductive health” [65].
CONTROL OF VIOLENCE AGAINST WOMEN	
Attitude towards wife beating	The percentage of women aged 15–49 years who believe a husband/partner is justified in hitting or beating his wife/partner for any of the following five reasons: argues with him, refuses to have sex, burns the food, goes out without telling him, or when she neglects the children [65].
Exposure to physical violence	The percentage of ever partnered women aged 15–49 years who were subjected to physical violence, sexual violence or both by a current or former intimate partner in the last 12 months [65].
Female homicide	Unlawful death inflicted upon a female with the intent to cause death or serious injury. This information was extracted from statistics by UN Office on Drugs and Crime based on national statistical systems through the annual United Nations Survey on Crime Trends and Operations of Criminal Justice Systems. The statistics reported numbers per 100,000 population [69].

### Data on decision-making status

Women’s decision-making status was measured using the proportion of currently married women aged 15–49 years who could make four decisions about: family visits, household purchases, their own health care, and sexual health (Table 1).

### Data on violence against women

Women’s status on exposure and tolerance of physical violence was measured by three variables: attitude of women towards wife-beating, exposure of women to physical violence and homicide of females (Table 1).

### Prevalence of ECC

The outcome variable was the prevalence of ECC. Data on ECC prevalence from 2007 to 2017 were obtained from the study by El Tantawi et al. [3]. These are data for children under 6 years of age who had one or more decayed, missing or filled primary tooth [70] split by age groups: 0 to 2 years, and 3 to 5 years. ECC prevalence data were extracted from the World Health Organization Country Oral Health Profile database, Web of Science, Scopus, Medline and Google Scholar databases using database-specific search terms. We also scanned articles published in local journals and government reports with no language filter. The retrieved country-data were used to calculate ECC prevalence per country by dividing the total number of children affected by ECC in each study by the total number of children examined and multiplying by100.

### Data analysis

General linear models were used to assess the relationship between outcome variables - ECC in 0 to 2 year olds and ECC in 3 to 5-year-olds - and three categories of explanatory variables - indicators of economic empowerment, indicators of decision-making ability, and indicators of women exposure to violence. The models were adjusted for the economic level of countries using the World Bank list of economies for 2017. Regression estimates (B), 95% confidence intervals (95% C.I.) and partial eta squared (η^2^) were calculated. SPSS version 23 (IBM Corp., Armonk, N.Y., USA) was used for statistical analysis. Significance was set at 5%.

### Ethical considerations

This study did not require ethical approval as the data used were publicly available blinded data.

## Results

### Global level indicators of women’s economic empowerment, decision-making ability, exposure to violence and ECC prevalence

Table 2 provides an overview of the data available for the study. It shows that the number of countries with available data varied: only a fifth of the 193 United Nation countries (20.2%, *n* = 39) had data about the percentage of females living below 50% of median income while 90.7% (*n* = 175) countries had data on homicides per 100,000 female. The data on prevalence of ECC for 3–5-year-olds was available in 89 (57.3%) countries and that of 0–2-year-olds was available in only 39 (23.8%) countries.

**Table 2:** Description of indicators and ECC prevalence among countries included in the study

Variables	Countries with available data (n)	Mean (SD)
Economic empowerment indicators		
Financial account	154	47.8 (31.6)
Education	76	50.9 (26.4)
Food insecurity	137	29.8 (23.2)
Living in slums (%)	60	55.7 (16.6)
Below 50% of median income	39	14.0 (55.6)
Women’s Decision-making indicators: Percentage of women participating in decisions related to:		
Family visits	57	71.3 (17.9)
Household purchase decisions	58	62.9 (19.3)
Own health care	58	66.1 (21.7)
Sexual health	44	47.6 (19.4)
Exposure to violence		
Attitude towards wife beating	95	33.2 (24.4)
Exposure to physical violence	68	14.9 (11.4)
Female homicide	175	2.9 (2.9)
ECC prevalence		
Among 0–2 year-olds	39	23.8 (14.8)
Among 3–5 year-olds	86	57.3 (22.4)

### ECC and women’s economic empowerment status

In the countries included in the analysis, 55.7% of women lived in slums, while 47.8% had financial institution accounts (Table 2). The associations between the five economic empowerment indicators and global prevalence of ECC are highlighted in Table 3. Countries with more females living under 50% of median income had a significantly higher prevalence of ECC for 3 to 5-year olds (B = 1.82, 95% CI = 0.12, 3.52). Meanwhile, though countries with higher percentages of educated women had higher ECC prevalence in both age groups, (η2 = 0.27 and 0.05) and those with higher percentages of females with food insecurity had lower ECC prevalence (η^2^ = 0.02 and 0.004), these relationships were not statistically significant.

**Table 3:** Relationship between economic empowerment of women and global ECC prevalence, controlling for country economic level

Variables	Prevalence of ECC in 0–2 years old			Prevalence of ECC in 3–5 years old		
	Countries with available data (n)	B (95% CI)	η^2^	Countries with available data (n)	B (95% CI)	η^2^
Financial account	37^a^	0.05 (−0.20, 0.30)	0.005	82^f^	−0.25 (− 0.50, 0.0001)	0.05
Education	15^b^	0.37 (−0.03, 0.78)	0.27	28^g^	0.18 (−0.16, 0.52)	0.05
Food insecurity	34^c^	−0.15 (− 0.58, 0.29)	0.02	73^h^	−0.10 (− 0.47, 0.27)	0.004
Living in slums	11^d^	0.16 (−0.52, 0.84)	0.04	19^i^	−0.25 (− 0.93, 0.43)	0.04
Below 50% of median income	17^e^	−0.23 (−2.03, 1.57)	0.006	32^j^	1.82 (0.12, 3.52)*	0.15

B: Regression estimates adjusted for country income, *CI* confidence interval, η^2^ partial eta squared, *: statistically significant at *P* <  0.05

Number of countries with different economic levels included in models, *LIC* low-income countries, *LMIC* low middle-income countries, *HMIC* high middle-income countries, *HIC* high-income countries

^a^LIC = 2, LMIC = 11, HMIC = 11, HIC = 15, ^b^LIC = 2, LMIC = 8, HMIC = 5, HIC = **0**, ^c^LIC = 2, LMIC = 11, HMIC = 7, HIC = 14, ^d^LIC = 2, LMIC = 7, HMIC = 2, HIC = **0**, ^e^LIC = 0, LMIC = 2, HMIC = 6, HIC = 9

^f^LIC = 5, LMIC = 19, HMIC = 25, HIC = 33, ^g^LIC = 6, LMIC = 13, HMIC = 9, HIC = **0**, ^h^LIC = 5, LMIC = 18, HMIC = 20, HIC = 30, ^i^LIC = 5, LMIC = 11, HMIC = 3, HIC = **0**, ^j^LIC = 0, LMIC = 2, HMIC = 9, HIC = 21

### ECC and women’s decision-making status

In the countries included in the study, up to 71.3% of women participated in decisions about visiting family and friends, 62.9% participated in major household purchase decisions, 66.1% participated in their own health care decisions and 47.6% made informed decisions about sexual relations and use of contraceptives (Table 2). Table 4 shows the association between the four decision-making indicators and global prevalence of ECC. Countries with higher percentage of women participating in their own health care decisions had higher prevalence of ECC for 0 to 2-year-olds (B = 0.85, 95% CI = 0.03, 1.67). Meanwhile, countries with higher percentage of women participating in decisions related to visiting family, relatives and friends had higher prevalence of ECC for 3 to 5-year-olds (B = 0.67, 95% CI = 0.03, 1.32).

**Table 4:** Relationship between decision-making ability of women and global ECC prevalence, controlling for country economic level

Variables	Prevalence of ECC 0–2 years old			Prevalence of ECC 3–5 years old		
	Countries with available data (n)	B (95% CI)	η^2^	Countries with available data (n)	B (95% CI)	η^2^
Family visits	9^a^	0.90 (−0.20, 2.02)	0.47	19^c^	0.67 (0.03, 1.32)*	0.25
Household purchase decisions	9^a^	0.80 (−0.20, 1.80)	0.46	19^c^	0.61 (−0.0004, 1.23)	0.23
Own health care decisions	9^a^	0.85 (0.03, 1.67)*	0.59	19^c^	0.49 (−0.09, 1.07)	0.18
Sexual health	5^b^	1.69 (−2.67, 6.05)	0.96	13^d^	−0.04 (−1.23, 1.14)	0.001

B: Regression estimates adjusted for country income, *CI* confidence interval, η^2^ partial eta squared, *: statistically significant at *P* <  0.05

Number of countries with different economic levels included in models, *LIC* low-income countries, *LMIC* low middle-income countries, *HMIC* high middle-income countries, *HIC* high-income countries

^a^LIC = 2, LMIC = 5, HMIC = 2, HIC = 0, ^b^LIC = 2, LMIC = 2, HMIC = 1, HIC = 0, ^c^LIC = 6, LMIC = 9, HMIC = 4, HIC = 0, ^d^LIC = 6, LMIC = 5, HMIC = 2, HIC = 0

### ECC and women’s exposure to violence

In the countries included in the study, 33.2% of women believed husbands were justified to beat their wives and 14.9% experienced physical/sexual violence during the previous year. In the 175 countries analyzed for killing of women, 2.9 in 100,000 females were unlawfully and intentionally killed (Table 2). Table 5 shows that there were no significant associations between the three indicators of women’s exposure to violence and global prevalence of ECC in countries included in the study.

**Table 5:** Relationship between women exposure to violence, suicide and global ECC prevalence after controlling for country economic level

Variables	Prevalence of ECC 0–2 years old			Prevalence of ECC 3–5 years old		
	Countries with available data (n)	B (95% CI)	η^2^	Countries with available data (n)	B (95% CI)	η^2^
Exposure to violence						
Attitude toward wife beating	15^a^	0.13 (−0.68, 0.93)	0.01	33^d^	0.05 (−0.40, 0.50)	0.002
Exposure to physical violence	14^b^	0.16 (− 2.84, 3.16)	0.002	34^e^	−0.52 (− 1.32, 0.29)	0.06
Female homicide	39^c^	0.19 (− 1.50, 1.89)	0.002	82^f^	0.15 (− 1.63, 1.94)	< 0.0001

B: Regression estimates adjusted for country income, *CI* confidence interval, η^2^ partial eta squared, *: statistically significant at *P* < 0.05

Number of countries with different economic levels included in models, *LIC* low-income countries, *LMIC* low middle-income countries, *HMIC* high middle-income countries, *HIC* high-income countries

^a^LIC = 2, LMIC = 8, HMIC = 5, HIC = 0, ^b^LIC = 2, LMIC = 3, HMIC = 2, HIC = 7, ^c^LIC = 2, LMIC = 11, HMIC = 11, HIC = 15

^d^LIC = 6, LMIC = 15, HMIC = 11, HIC = 1, ^e^LIC = 5, LMIC = 7, HMIC = 5, HIC = 17, ^f^LIC = 6, LMIC = 20, HMIC = 23, HIC = 33

## Discussion

This is the first study highlighting how women’s empowerment status may be a risk indicator for ECC at a global level. The study findings suggest that the prevalence of ECC for 3 to 5-year-olds was higher where the proportion of women with < 50% of median income was greater and where the proportion of women participating in decisions about socializing was higher. We also observed that the higher the proportion of women who participated in decisions about their own health care, the higher the prevalence of ECC in 0 to 2-year-olds. No associations were identified for women’s exposure to violence and ECC prevalence. Our results, therefore, partly support the study hypothesis.

We avoided the use of a summative index to describe women’s empowerment because this may have obscured item-level distinctions and decrease the predictive value of the construct [24]. The results of our study justified this decision as we noticed that the prevalence of ECC was higher where one indicator of economic empowerment; females living under 50% of median income was worse and where decision-making status was better. Empowerment is a multidimensional concept and its domains are not necessarily correlated [28]. These domains are moderated by different social factors and their pathways for affecting women’s empowerment and ECC may differ.

We found conflicting associations between the prevalence of ECC and indicators of women’s economic empowerment. On one hand, higher prevalence of ECC was observed where there were higher proportion of women with low income suggesting that financial empowerment was associated with less ECC as reported in prior studies [71–74]. On the other hand, the prevalence of ECC was higher where there were more educated women and food security. We feel this direct relationship between economic empowerment and the prevalence of ECC is because of women’s empowerment is envisioned as their inclusion into paid labor force [75]. Access to maternity leave may enable women to support healthy eating habits and provide oral health care to infants and toddlers [76] thereby reducing their risk for ECC. On return to work, maternal care may diminish if the mother’s attention becomes divided between the child and work [77, 78], negatively affecting dietary and oral hygiene habits, and increasing the risk of ECC especially for older children as the result of this study suggests.

Women’s economic empowerment frameworks that value paid work and undermine the value of domestic care may have a negative impact on child oral health. Economic empowerment policies and programs may need to recognize the needs of women to have fewer hours of work outside home to enable them to care for children, and provide financial support for this home care role. The prior observation that empowerment was associated with less ECC may likely be a result of improved personal hygiene practices rather than conscious decisions about time and money allocation [79]. In addition, lower prevalence of ECC may result with changes in social norms that promote less gendered division of child caretaking responsibilities [80]. Further studies are required to evaluate how maternal economic empowerment impacts pre-school child’s risk for ECC at family, community and country levels.

We also observed that the prevalence of ECC may be higher with two indictors of decision-making. We assume this may be because women with autonomous decision-making powers have greater mobility and better social networking that may result in higher exposure and access of older preschool children to processed cariogenic food [24]. On the other hand, women who are empowered to make decisions about their health may choose to plan pregnancies further apart [16] and increase the duration and frequency of breastfeeding for younger preschool children. Prolonged breastfeeding beyond 24 months of age is a risk factor for ECC in young pre-school children [81, 82]. Nevertheless, this explanation of our study findings needs further exploration.

We postulated that reduced exposure to violence arising from women’s economic and social empowerment may decrease the prevalence of ECC. Violence leads to mental health challenges such as anxiety, depression, and post-traumatic stress disorder [83], which reduce maternal competency to attend to children’s health including oral health [84]. Our findings did not support our postulation and did not agree with the results of few studies demonstrating negative impact of exposure of women to violence on child health [85–87]. This lack of association reported in our current study may be attributed to the relatively low prevalence of violence practices (exposure to physical/sexual violence and female homicide), which may partly explain the weak and non-significant association.

While our study provided new information on risk indicators for ECC, we call for caution when interpreting the findings because of few limitations. First, we were able to adjust only for country’s economic level. Mutual adjustment for all the factors in the present study was not possible because of data unavailability. Adjusting for one more factor beyond the economic level reduced the sample size in some cases to zero country. We chose to provide an insight into potential relationships between ECC prevalence and indicators of women empowerment using simplified statistical models with minimal adjustment rather than using more comprehensive modeling technique that require data which may not be available in the foreseeable future. Also, macro-level and country-level factors affect the ability of individuals to maintain their and their family members’ health. For example, regardless of the economic empowerment and decision-making ability of a woman, health services can only be accessed and utilized if available. However, we were unable to control for such country-level factors. In addition, we used data from global surveys such as the DHS that generally target non high-income countries (HICs). Because of this, our findings about the association between decision-making ability and ECC prevalence cannot be generalized to HICs. We also had few country-level data on ECC for children 0 to 2 years old, with implications for making findings for this age group not globally representative. ECC is a multifactorial disease and risk factors may differ between cultures with different practices, beliefs and traditions suggesting the need for country-specific studies prior to adoption of our study findings to guide country-level policy formulation and program development. Evidence based on individual-level data collected about the relationship between women empowerment and ECC prevalence in preschool children may support or refute our findings and thus, provide evidence for what our study suggests.

## Conclusion

Despite these limitations, our study provided provisional perspective on the complex relationship between women gaining agency through economic empowerment and autonomous decision-making and the risk for ECC. Our study suggests that some measures of women empowerment may be associated with higher risk for ECC and that as women gain “power” they become victims of the pitfalls of modern western society that requires women to do more for economic comfort. This struggle may result in the relegation of homecare duties as mothers may resort to quick meals, packaged meals or sugary snacks. The study findings therefore suggest the need for safeguards for the oral health of children especially in societies with transitional economies that are working towards empowering women. Further studies are required to explore how the gaining of agency by women can reduce the risk for ECC through the institution of supportive policies that do not undermine the economic empowerment of women through mechanisms that positively influence children’s caries risk behavior or through the use of resources (money and time) to acquire healthy food and oral health habits.

## Supplementary information

**Additional file 1.** Country level data on prevalence of ECC (0–2-year-olds and 3–5-year-olds), women economic empowerment, women decision-making (family visits, household purchases, their own health care, and sexual health) and women exposed to violence (attitude of women towards wife-beating, exposure of women to physical violence and homicide of females).

## Data Availability

All study materials have been submitted with the manuscript.

## Abbreviations

B: Regression estimates
CI: Confidence Interval
ECC: Early childhood caries
HIC: High-income countries
HMIC: High middle-income countries
LIC: Low-income countries
LMIC: Low middle-income countries
PR: Prevalence Ratio
SD: Standard Deviation
η^2^: Partial eta squared
